# Assessment of surface treatment methods for strengthening the interfacial adhesion in CARALL fiber metal laminates

**DOI:** 10.1038/s41598-024-81777-1

**Published:** 2024-12-28

**Authors:** Madhusudhan Balkundhi, Satish Shenoy Baloor, Gururaj Bolar

**Affiliations:** 1 Department of Mechanical and Industrial Engineering, Manipal Institute of Technology, Manipal Academy of Higher Education, Manipal, 576104 Karnataka India; 2 Department of Aeronautical and Automobile Engineering, Manipal Institute of Technology, Manipal Academy of Higher Education, Manipal, 576104 Karnataka India

**Keywords:** Fiber metal laminates, Surface treatment, Interfacial adhesion, Peel strength, Shear strength, Surface characterization, Composites, Mechanical engineering

## Abstract

Metal and polymer interface bonding significantly influences the mechanical performance of fiber metal laminates (FMLs). Therefore, the effect of surface treatments (mechanical abrasion, nitric acid etching, P2 etching, sulfuric acid anodizing (SAA), and electric discharge machine (EDM) texturing) carried on aluminum 2024-T3 alloy sheets was evaluated considering surface morphology, surface topography, and surface roughness. Further, the influence of surface treatments on interfacial adhesion strength and failure mode between the aluminum alloy and carbon fiber prepreg was investigated. The surface treatments increased the surface roughness of the aluminum substrates. Surfaces treated using SAA, nitric acid, and P2 etchant showed improved wettability, while mechanically abraded and EDM textured substrates showcased hydrophobic behavior. The selected surface treatments significantly affected interfacial adhesion between the epoxy polymer and aluminum alloy. SAA and EDM texturing greatly enhanced the interfacial peel strength of FMLs. In the case of interfacial shear strength, EDM textured substrate showed superior performance, followed by SAA. Moreover, untreated and mechanically abraded specimens exhibited weaker bonding and adhesive failure at the aluminum-epoxy interface, whilst chemical treatments resulted in mixed model failure. EDM textured surface underwent cohesive failure, while a dominant mixed mode failure and fiber adhesion were observed in the SAA-treated specimen.

## Introduction

Composite materials like fiber metal laminates (FMLs) are developed by stacking polymer composites and light metals like aluminum, magnesium, or titanium, combining the qualities of both composites and metals^1^. FMLs have excellent fatigue resistance, impact resistance, anti-corrosion behavior, and damage tolerance, among many other attributes^2^. As a result, they find wide applications in the aviation, automobile, and defense industries^1,2^. The most widely used FMLs in industries are glass aluminum-reinforced epoxy (GLARE) and aramid-reinforced aluminum laminates (ARALL). Nonetheless, FMLs comprising aluminum and carbon fiber-reinforced aluminum laminates (CARALL) have drawn attention lately because of their superior tensile qualities, low density, and high modulus of elasticity^3,4^. FMLs are fabricated by curing the constituent materials under specific temperatures and pressures based on the selected polymer material, forming a bond at the interface. However, the thermo-mechanical characteristics of the FML constituents strongly impact the bond strength. The difference in the elastic–plastic properties and thermal expansion coefficient of the constituent metal and composite can result in residual stresses at the interface, facilitating delamination and fracture, especially in FMLs subjected to cyclic thermal loading^5,6^. Therefore, strengthening the metal-polymer bonding is crucial to increase the interfacial strength and enhance the durability and reliability of FMLs^7,8^.

Several pre-surface treatment methods are employed to improve the metal-composite interfacial adhesion. These pre-surface treatment procedures fall into one of three categories: thermal, chemical, or mechanical^9^. Mechanical treatments like mechanical abrasion, grit blasting, sandblasting, etc., increase surface roughness by altering surface morphology^10^. The enhanced adhesion behavior of mechanical methods can be attributed mainly to increased surface energy and mechanical interlocking between metals and composites^8,11^. Studies have found that the interfacial adhesion between metals and composites like carbon fiber-reinforced plastics (CFRP) and glass fiber-reinforced plastics (GFRP) can be strengthened by increasing the surface roughness^12,13^. Recently, interfacial shear failure in GLARE was investigated considering the sandpaper treatment. However, the mechanical abrasion increased the macro roughness, thereby increasing the contact angles and lowering the surface wetting area, surface energy, and shear adhesion strength^14,15^.

Researchers have employed chemical treatment methods to improve the interfacial adhesion behavior of aluminum surfaces. Etching using the forest products laboratory (FPL) method promoted strong interfacial adhesion, increasing the flexural strength of metal laminates made of basalt fiber^15^. Compared to the sanded specimens, chemical treatment with sodium hydroxide and nitric acid greatly enhanced the wettability and roughness^14,16^. Samples subjected to combined alkaline and acid treatment methods exhibited a higher energy release rate and increased interfacial fracture toughness^17^. A recent study evaluated the adhesion behavior and ILSS of specimens subjected to mechanical abrasion and chromic acid anodizing (CAA) method, where CAA-treated samples showed oxide layer deterioration under harsh environmental conditions^18,19^.

Newer methods like plasma^20,21^, laser^22,23^, and sol–gel treatment^24,25^ are also used to improve the surface characteristics for better adhesion. Plasma treatment increases the surface polarity by causing surface oxidation and removing the carbonaceous components, enhancing the surface wettability. However, when the scan speed or number of scans increased, the wettability of the aluminum surface declined^20,21^. Laser ablation produced higher roughness with the increased number of laser passes. Laser-textured surface textures outperformed chemically treated specimens in terms of shear strength in Mode I and Mode II loading conditions by 40% and 110%, respectively, while fracture toughness increased by 52% 22, 23. However, the increasing number of passes reduced the lap shear strength due to air entrapment into the laser-ablated zones^22^. The sol–gel coating offered better adhesion by improving the affinity of the surface to create chemical bonds between the metal and composite interface^24,25^.

The reviewed literature indicates that metal surface treatments help enhance the adhesive bonding quality between the metal and composite layers of FML. A few studies have examined how surface treatment techniques affect the adhesively bonded aluminum. However, studies evaluating the influence of various surface treatments on the peeling strength (Mode I loading) and the shear strength of the adhesive bond (Mode II loading) between the metal and composite layers in CARALL are limited. Furthermore, there is a lack of research on the effects of electric discharge machining (EDM) based texturing on the metal surface characteristics and its influence on the interfacial strength between the metal and composite layers of CARALL FML. Therefore, the current study evaluates the effect of different surface treatments (mechanical abrasion, nitric acid etching, P2 etching, sulfuric acid anodizing (SAA), and EDM texturing) on the surface morphology, surface topography, and surface roughness characteristics of aluminum 2024-T3 alloy used to fabricate CARALL. The impact of different surface treatment methods on the interfacial strength and fracture mechanisms in aluminum/CFRP laminates subjected to Mode I and Mode II loading conditions was investigated using the T-peel and shear peel tests. The methodology and the outcomes of the experimental work are explained in the following sections.

## Materials and methods

### Materials

The metal substrate used in the study was solution heat-treated and cold-worked aluminum 2024-T3 alloy with a nominal thickness of 0.5 mm, ultimate tensile strength of 480 MPa, and modulus of elasticity of 72.4 GPa (Mangaldeep Metals and Alloys). A carbon fiber prepreg system consists of unidirectional carbon fibers (200 GSM) and a thickness of 0.2 mm with A-45 epoxy resin (resin content of 38 ± 3%) (Bhor Chemicals and Plastics Private Limited). The prepreg system is designed for autoclave and hot press curing methods and offers improved fracture toughness, strength, and adhesive properties.

### Substrate preparation

In the paper, five different surface treatments, i.e., mechanical abrasion, nitric acid etching, P2 etching, SAA, and EDM texturing, were compared to study the influence of surface characteristics on adhesive strength at the interface between aluminum and carbon fiber/epoxy. At first, specimens were rinsed using deionized water and cleaned using a forced air supply. The aluminum specimens were dried in a convection oven for 30 min at 40 °C. Specimens after drying were further subjected to ultrasonic cleaning for 10 min using an acetone ultrasonic bath at 40 Hz and dried at room temperature. The cleaned specimens were subjected to five different surface treatments, the details of which are provided in the subsections.

#### Mechanical abrasion

The mechanical abrasion was performed on the metal surface using 60 grit size abrasive paper. After the abrasion process, the metal surfaces were cleaned using deionized water. The abraded specimens were degreased using a sodium hydroxide (NaOH) solution of 30% concentration by weight^20^. After degreasing, aluminum specimens were rinsed in deionized water and dried in hot air to remove any remaining moisture.

#### Chemical treatments

Chemical treatments in the form of nitric acid etching and P2 etching were considered for the study. Before the chemical treatments, surfaces were degreased using 30% NaOH solution for 30 s. In the nitric acid etching method, the specimen was treated using nitric acid (HNO_3_) at room temperature with 30% concentration by volume for 60 s^14^. In the P2 etching method, the P2 etching solution was prepared by mixing 15% of ferric sulfate (Fe_2_(SO_4_)_3_) and 37% of sulphuric acid (H_2_SO_4_) in 48% of deionized water^26^. P2 etching was performed at 60 °C to 65 °C for 10 min. After the chemical treatments, specimens were rinsed in deionized water and dried using hot air at 40 °C.

#### Sulphuric acid anodizing

In the Sulphuric Acid Anodizing (SAA) method, specimens were pre-treated with 30% sodium hydroxide solution for 30 s in order to clear the oil and greasy elements from the surface. After degreasing, the specimens were rinsed in deionized water and dried. Then, the specimens were pre-treated with a 30% NaOH solution for 30 s. Finally, the specimens were anodized for 30 min in 0.5 M sulphuric acid^11^. The solution was continuously stirred using a magnetic stirrer at a lower speed. Finally, the treated aluminum specimens were rinsed in water and dried using hot air for an hour at 40 °C.

#### EDM texturing

Surface texturing using the EDM process was carried out on a Sparkonix ZNC 50A. EDM experiments were conducted by setting the current at 4 A, pulse-on time of 150 µs, and pulse-off time of 70 µs. DEF 92, a low-viscosity commercial EDM fluid, was used as the dielectric. The EDM textured specimens were annealed to relieve any accumulated stresses. The EDM textured specimens were cleaned and degreased with a 30% NaOH solution for 30 s to remove any remaining oil traces on the surface. The degreased surfaces were rinsed in deionized water and dried in hot air at 40 °C.

### Surface characterization

The substrate surface was analyzed for surface morphology, surface topography, surface roughness, contact angle, and surface energy. The untreated and treated substrate surfaces, before and after failure, were analyzed using a scanning electron microscope (SEM) (Carl Zeiss EVO–MA18), while the surface chemistry was studied using energy dispersive spectroscopy (EDS). A non-contact optical profilometer (Bruker – Contour) was used to obtain 3D surface tomographs and surface roughness. In the study, the average roughness value was taken into account after three measurements were made. A contact angle analyzer (Holmarc HO–ED–M–01) was utilized to measure the contact angle of the water with the aluminum substrate. A 5 µl of deionized water was deposited on the test surface to measure the contact angle. Surface energy is the key contributor to adhesion quality. It is the surplus energy that is available at the surface compared to bonding energy within the material that keeps the material intact. For each surface treated, the surface energy was calculated as a function of contact angle using the Owens–Wendt-Rabel-Kaelble equation given by:

1 $$\gamma_{l} (1 + \cos \theta ) = 2(\sqrt {\gamma_{s}^{d} \gamma_{l}^{d} + \gamma_{s}^{p} \gamma_{l}^{p} } )$$

where, ${\gamma }_{l}$= surface tension of the liquid, $\theta$= contact angle of liquid, ${\gamma }_{s}^{d}$= dispersive component of solid, ${\gamma }_{l}^{d}$= dispersive component of liquid, ${\gamma }_{s}^{p}$= polar component of solid, and ${\gamma }_{l}^{p}$= polar component of liquid^27^.

### T-peel and lap-shear specimen preparation

In the current investigation, two fiber/epoxy prepreg layers were positioned between two aluminum alloy sheets. After the assembly, specimens were vacuum bagged and autoclave cured (see supplementary information, Fig. S1(a)). The specimens were cured according to the curing cycle (see supplementary information, Fig. S1b) by initially heating to 90 °C by increasing the temperature at a rate of 2 °C/min. Following an isothermal dwelling of 30 min, the temperature was raised to 120 °C at a rate of 1.5 °C/min and maintained for a dwell period of 60 min. At the end, specimens were cooled to 30 °C at 3 °C/min.

### T-peel and lap-shear tests

**Fig. 1:**
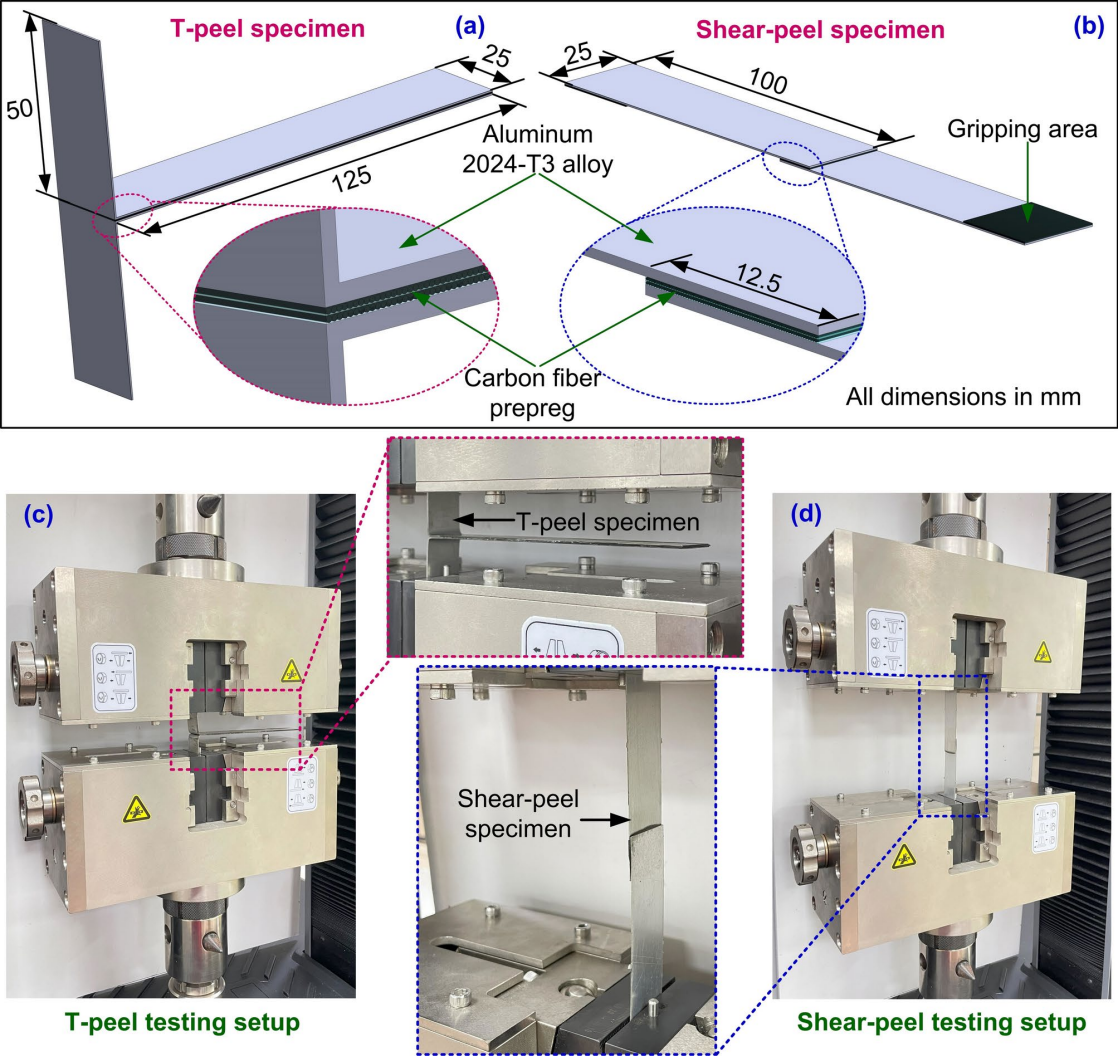
(**a**) T-peel test specimen, (**b**) Lap-Shear test specimen, (**c**) Specimen under T-peel test setup, (**d**) Specimen under lap-shear test setup.

The interfacial peel resistance (Mode I loading) and interfacial shear strength (Mode II loading) were assessed for untreated and surface-treated specimens. Five tests were conducted for each surface treatment, and the average values were considered in the study. Figures 1(a, b) show the geometries and dimensions of T-peel and shear-peel test specimens. To determine the adhesion characteristics under Mode I loading, the T-peel test was performed by applying the load perpendicular to the bonding plane in accordance with the ASTM D-1876 standard. The crosshead of a universal testing machine (MTS Exceed) was moved at a speed of 5 mm/s to perform T-peel tests (see Fig. 1c). Equation (2) was utilized to compute the peeling strength.

2 $${\text{Peeling Strength = }}\frac{{\text{Average peeling force}}}{{\text{Bonding area width}}}$$

The lap shear test was performed to study the adhesive behavior in Mode II loading, where the load was applied parallel to the bonding plane. The lap shear testing following ASTM D-3163 was carried out at a crosshead speed of 2 mm/s (see Fig. 1d). Equation (3) was used to compute the shear adhesion strength.

3 $$Shear \, Adhesion \, Strength \, = \, \frac{{P_{{_{S} }} }}{W \times L}$$

where *P*_*S*_ is the peak shear force, *W* is the width of the substrate, and *L* is the length of the bonding area.

## Results and discussion

### Surface morphology and roughness

**Fig. 2:**
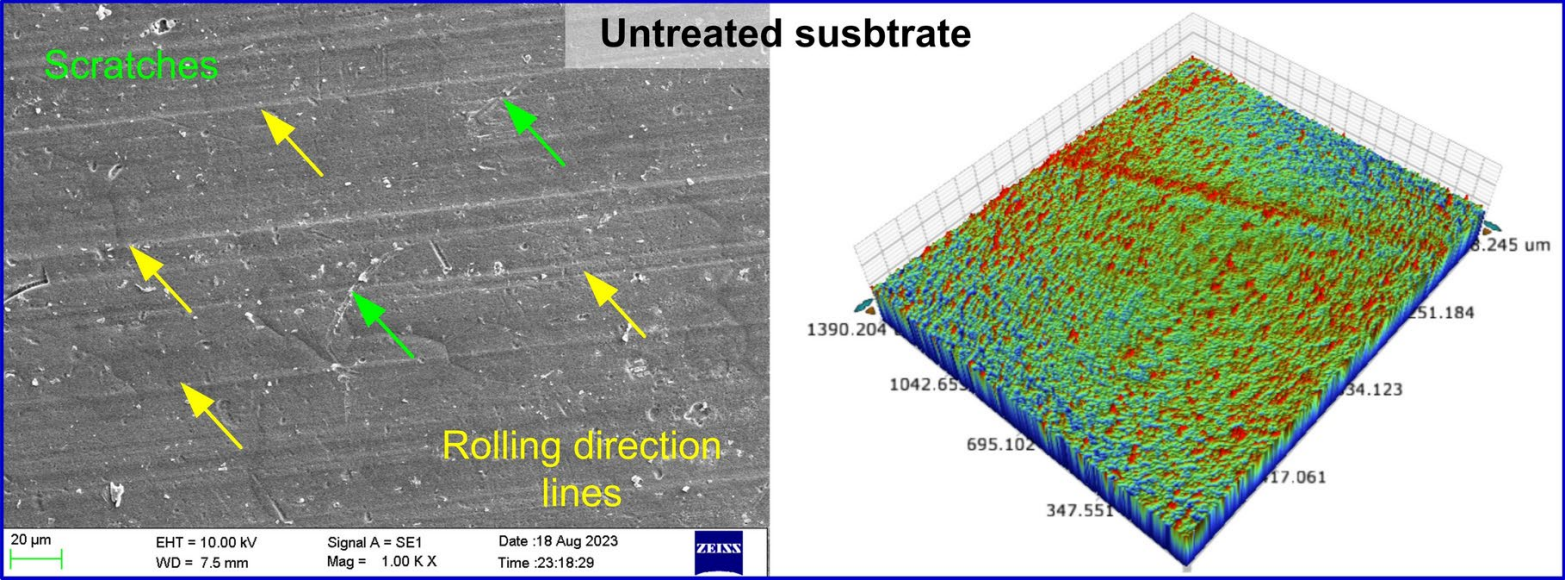
Surface morphology and 3D surface topography of untreated substrate.

**Fig. 3:**
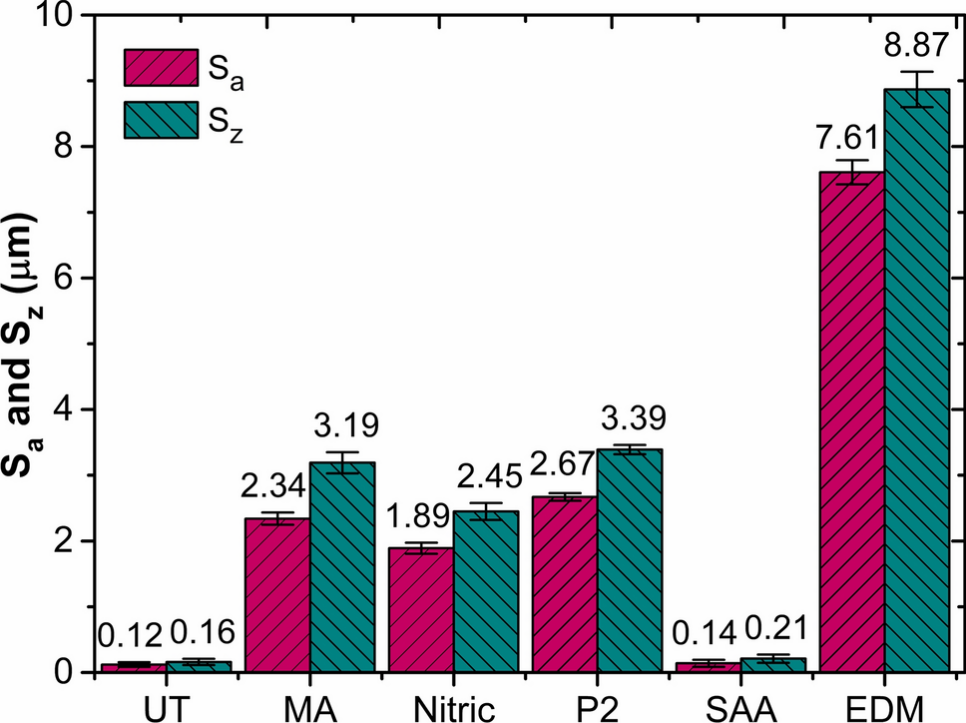
Average roughness values for untreated and treated aluminum substrates.

**Fig. 4:**
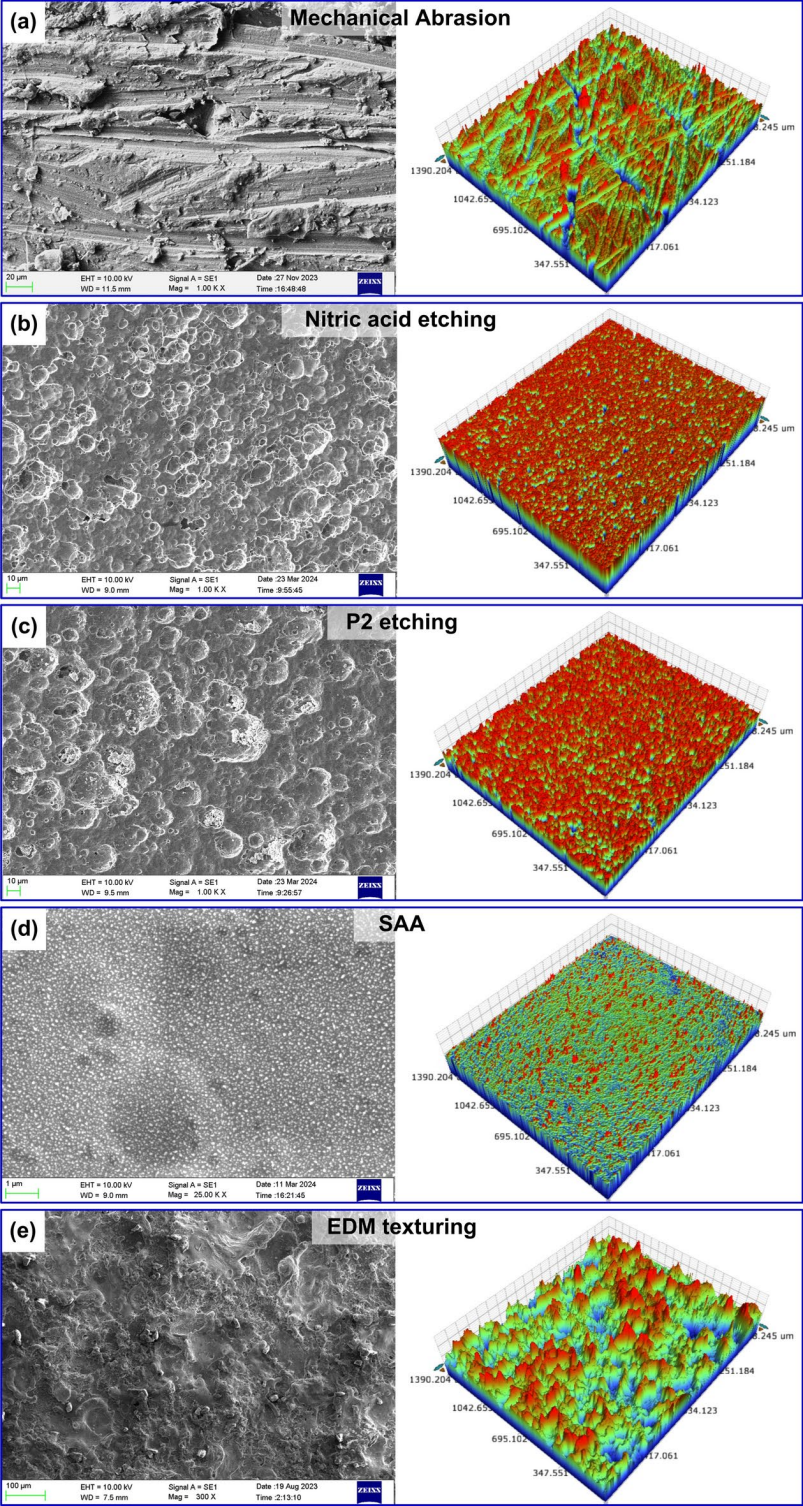
Surface morphology and 3D surface topography of substrates subjected to (**a**) Mechanical abrasion, (**b**) Nitric acid etching, (**c**) P2 etching, (**d**) SAA, (**e**) EDM texturing.

The aluminum substrates subjected to various surface treatments were analyzed considering the surface morphology and topography. Figure 2 shows the untreated and degreased surface, which is relatively smooth with lower *S*_*a*_ and *S*_*z*_ values (Fig. 3). The surface topography and morphology of the surface subjected to mechanical abrasion are depicted in Fig. 4a. The abraded surface showed a rougher appearance, indicating a notable morphological alteration compared to the untreated surface. The mechanically abraded substrate surface comprises of randomly oriented uneven grooves and ridges, with a higher *S*_*a*_ and *S*_*z*_ of 2.34 µm and 3.19 µm values.

Figure 4b shows the surface morphology and 3D topography of the aluminum surface after nitric acid treatment. A closer observation evidenced the presence of micro-scale cavities attributed to the chemical treatment. The aluminum 2024-T3 alloys consist of copper, magnesium, and manganese as its main alloying elements. The aluminum matrix is softened during chemical etching, making elements like copper, magnesium, and manganese susceptible to chemical dissolution. The alloying elements get dislocated from the softened aluminum matrix, creating micro-scale pores on the surface^11^. The micro-scale pores give the metal surface an irregular appearance and act as potential sites for anchoring the adhesive to bond. Moreover, *S*_*a*_ and *S*_*z*_ of 1.89 µm and 2.45 µm were measured (see Fig. 3), indicating a 1400-fold increase compared to the untreated specimen. Figure 4c depicts the surface morphology of an aluminum alloy sheet treated with P2 etchant. Similar to nitric acid etching, P2 etching resulted in greater roughness due to the formation of micro-scale pores. The microscale pores were formed because of the dissolution of the aluminum matrix and alloying elements during the etching process. The peaks and valleys increase the surface roughness and surface area, thus the bonding strength. The *S*_*a*_ and *S*_*z*_ measured for the P2 etched specimen were around 2.67 µm and 3.39 µm, respectively (see Fig. 3).

Figure 4d shows the surface morphology and topography of aluminum substrates treated with SAA. Nano-scale pores and micro-scale pits make up the majority of the surface. The nano-porous structure is attributed to the alumina layer developed on the aluminum surface. Aluminum metal undergoes chemical reactions during anodizing in two stages. The evolution of oxygen is due to the decomposition of water (2H_2_O = 4H + O_2_ + 4 e-) and the oxidation of aluminum (4Al + 3O_2_ = 2Al_2_O_3_)^28^. The alumina thus formed deposits on the anode (aluminum) surface. Meanwhile, the micro-pits on the substrate surface result from the etching treatment. The copper in aluminum alloy exists as a solid solution and CuAl_2_. In the presence of the anodizing solution, due to the electrochemical activity, galvanic corrosion occurs between aluminum and CuAl_2_, resulting in material removal and micro-pit formation. The magnitude of *S*_*a*_ and *S*_*z*_ measured for the SAA substrate was 0.14 μm and 0.21 μm (see Fig. 3). It has to be noted that the size of the pores (in nanometres) is beyond the detection limit of the profilometer. Although the formation of nano-pores contributed very little to the increase in surface roughness, the nano features helped increase the surface area.

The surface morphology of the EDM textured surface shown in Fig. 4e indicated a rougher appearance. During EDM, the material undergoes melting and evaporation, resulting in the formation of craters. Closer inspection revealed a coral reef-like surface due to overlapping craters formed due to the constantly changing discharge position during the machining operation. Additionally, a recast layer transpired on the machined surface due to material melting resulting from rapid thermal cycles and re-solidification during cooling. As a result, a significant enhancement in the surface roughness was noted after EDM texturing. The recorded maximum values of *S*_*a*_ and *S*_*z*_ were 7.61 μm and 8.87 μm, an increase of 6241% and 5443%, respectively, compared to the untreated substrate (see Fig. 3).

### Surface chemical composition

Table 1 shows the chemical composition of the untreated and treated specimen surface, while the EDS spectra obtained by area scans of the pre-treated surfaces can be visualized from supplementary information, Fig. S2. The untreated and mechanically abraded specimens show similar chemical composition and slightly increased oxygen content. The increase can be attributed to the oxidation phenomenon. In comparison, the chemically treated specimens showed a different trend. As seen from Table 1, an increase in oxygen is noted, which is attributed to the formation of an oxide layer on the surface. Additionally, the amount of copper and magnesium decreased in the two chemically treated surfaces due to chemical dissolution. The SAA specimen exhibited a significantly higher amount of oxygen. This is attributed to the formation of the aluminum oxide layer during the anodizing process. However, other elements were detected in lower amounts. The reduction may be linked to the deposition of the oxide layer, which masks the presence of these elements in the base material. In the case of EDM texturing, the molten material ejected during the machining process reacts with the oxygen present in the dielectric to form a recast oxide layer upon deposition and cooling. As a result, the EDM textured surface showed increased oxygen content and reduced aluminum content. Additionally, an increased presence of copper was detected on the EDM machined surface, which is ascribed to the deposition of the material from the copper tool due to the tool wear. As noted, the oxide layer formed during the SAA treatment was beneficial as it helped increase the surface energy. Similarly, the dissolution of elements like copper and magnesium during chemical treatments increased the surface porosity, resulting in increased surface energy. On the other hand, the reduced surface energy exhibited by EDM textured specimens indicates that surface chemistry has a limited influence on wettability. Accordingly, surface roughness is more influential than surface chemistry in determining the wettability of the EDM textured surfaces.

**Table 1:** Elemental composition of untreated and treated specimens.

Surface Treatment	Al	Mg	O	Si	Ti	Cr	Mn	Fe	Cu	Zn	Surface energy
Untreated	90.7	1.5	2	0.2	0.2	0.6	0.9	1.3	2.4	0.2	35.3
MA	90.1	1.1	4.5	0.1	0.2	0.5	0.6	1.3	1.5	0.1	30.2
Nitric	88.5	0.9	6.4	0.2	0.3	0.6	0.7	1.1	1.2	0.1	40.1
P2	87.1	0.7	9.8	0	0.3	0.5	0.5	0.6	0.3	0.2	43.9
SAA	41.8	0.9	54.7	0.1	0.1	0.4	0.4	1.3	0.1	0.2	62.3
EDM	66.5	1.3	25.6	0.5	0.2	0.4	1.1	1.2	2.8	0.4	20.1

### Surface energy and contact angle

**Fig. 5:**
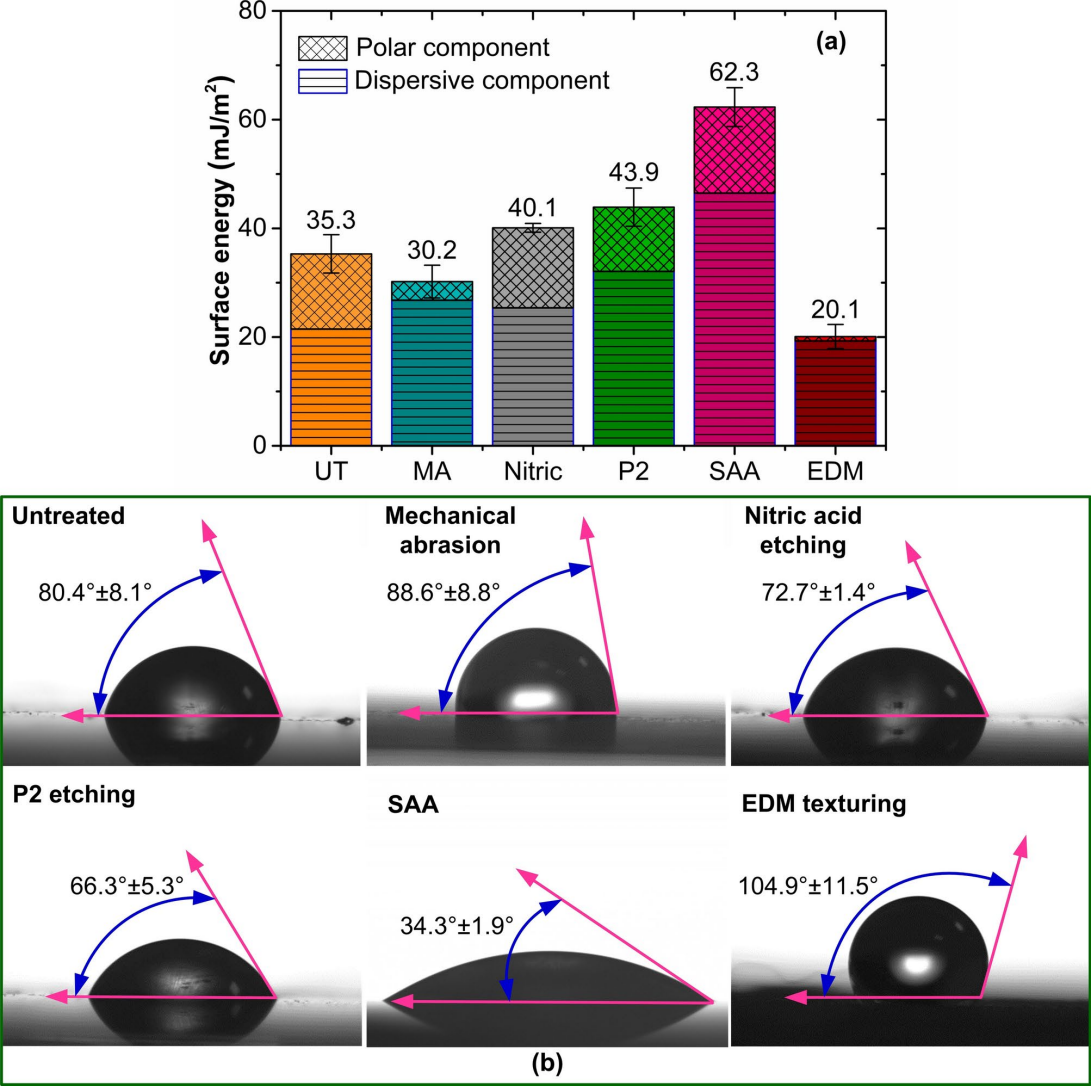
(**a**) Surface free energy of substrates subjected to different treatments, (**b**) Contact angle of the metal substrates.

Superior interference bonding can be obtained by increasing the wettability and surface energy of the metal. A solid surface exhibits wetting when its free energy rises. The total surface free energy comprises of dispersive and non-dispersive (polar) components. Both components are essential in predicting the wetting behavior. However, the high dispersive component favors wetting by nonpolar liquids (oil, gasoline, etc.), and the high polar component enhances wetting with polar liquids like water and epoxy^29^. Figure 5a displays the total surface energy of the different surface-treated specimens. The untreated surface exhibited a total free energy of 35.3 mJ/m^2^, with a dispersive component of 21.51 mJ/m^2^ and a polar component of 13.76 mJ/m^2^. In comparison, lower surface energies of 35.3 mJ/m^2^ and 20.1 mJ/m^2^ were observed in specimens subjected to mechanical abrasion and EDM texturing. Additionally, the polar components of the mechanically abraded (3.4 mJ/m^2^) and EDM textured (0.8 mJ/m^2^) specimens were very low, indicating curtailed wetting capability by polar liquids like water and epoxy. In comparison, higher surface energies of 40.1 mJ/m^2^, 43.9 mJ/m^2^, and 62.3 mJ/m^2^ were achieved in nitric acid-treated, P2 etched, and SAA-treated specimens. The polar components were significantly higher for the chemical treatments, with a magnitude of 11.8 mJ/m^2^, 14.7 mJ/m^2^, and 15.8 mJ/m^2^ observed for P2, nitric acid etching, and SAA treatment, indicating the enhanced capability of chemical and SAA treatments to form chemical bonds and increase the strength of the metal–composite interface.

Figure 5b shows the average contact angles between water and aluminum surfaces before and after surface treatments. The untreated surface exhibited an average contact angle of 80.4°. In comparison, specimens subjected to mechanical abrasion and EDM texturing showed a larger contact angle. The abrasion-induced grooves and large-sized craters act as sites for air entrapment. The surface becomes hydrophobic due to the air trapped in the craters and grooves, creating a micro-geometric barrier that keeps water droplets from penetrating the rough surface asperities^30^. Meanwhile, the contact angle for chemically and electro-chemically treated surfaces reduced significantly (see Fig. 5b). The three treatments generated micro or nano-pores on the aluminum surface. The reduction in pore size helped improve the capillary wetting, enabling the liquid to infiltrate the generated pores. It should be observed that the surface energy is dependent on the pore density. The surface energy increased with the increase in pore density. Accordingly, SAA-treated surfaces with higher pore density could spread the liquid to a larger area, enhancing surface energy and wettability.

### T-peel test

**Fig. 6:**
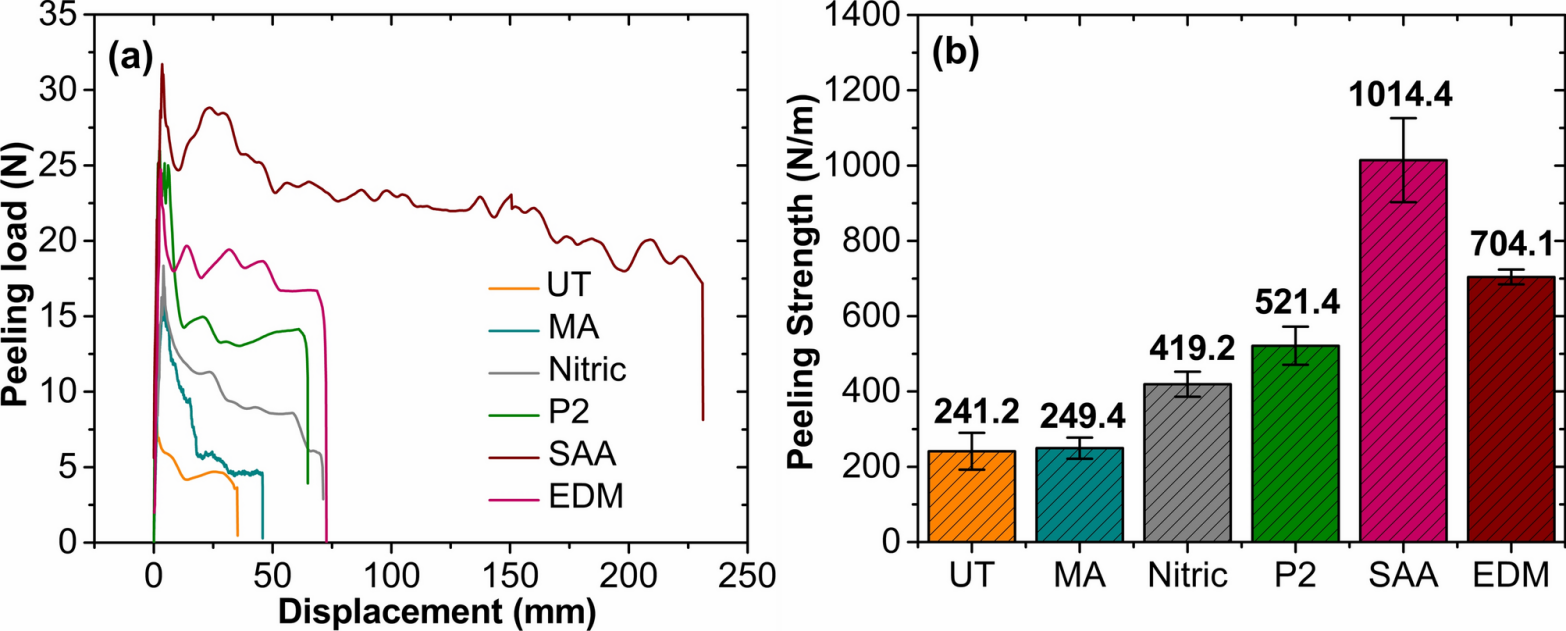
(**a**) Load–displacement curve for T-peel test, (**b**) Peeling strength for untreated and treated specimens.

Figure 6a displays the load–displacement curves obtained for the untreated and surface-treated specimens. The untreated specimen showcased a lower maximum peel load and load–displacement capacity than the treated specimens. Figure 6b exhibits the mean peel strength for the untreated and surface-treated specimens. In comparison to the treated specimens, untreated specimens exhibited lower peel strength. Lower strength characteristics are attributed to the limited surface roughness and wetting capability. The lower roughness of the untreated aluminum substrate (see supplementary information, Fig. S3 a) failed to provide sufficient mechanical interlocking capability and adhesive wettability, resulting in poor interfacial peel strength. In comparison, the aluminum specimens subjected to various surface treatments significantly influenced the peel strength.

Furthermore, Fig. 6b shows that the peel strength increased after surface treatments. The specimens subjected to mechanical abrasion showed a meager 3.4% increase in the peeling strength compared to the untreated specimens. Abrasion using silicon carbide (SiC) particles produced grooves on the substrate surface. However, the mechanically abraded specimens exhibited lower peeling strength than the chemical and electrochemical treatments. The scratches generated using an abrading medium are in the form of wide grooves (see supplementary information, Fig. S3(b)). The epoxy resin penetrating the grooves fails to create a strong mechanical interlock with the aluminum substrate, resulting in a weak interface and lower peel strength. In comparison, the peeling strength of specimens treated by chemicals (nitric acid etching and P2 etching) was increased by 73.9% and 116.2%, respectively. Adhesion enhancement is attributed to improved wettability, formation of rougher surfaces, and removal of surface contaminants. Micro-pores formed on the substrate surface during nitric acid etching serve as sites for epoxy intrusions and mechanical anchoring (see supplementary information, Fig. S3 c), thereby creating interlocks at the micro-scale. Furthermore, the micro-pore density on the P2 etched substrate surface (see supplementary information, Fig. S3(d)) was higher than the nitric acid-treated substrate. Accordingly, the number of interlocks formed per unit surface area increases. As a result, P2 etched specimens displayed a 24.4% increase in peeling strength compared to nitric acid-treated specimens. The aluminum substrate after SAA exhibited a 320.6% increment in strength compared to the untreated specimen. This significant improvement is attributed to the nano-scale morphology exhibited by the deposited oxide layer (see supplementary information, Fig. S3 e). The cured epoxy resin is embedded inside the nano-pores in the form of pins, thus enhancing the mechanical interlocking. When the external peeling load is applied, the pin-like structures at the interface reduce the stress concentration at the crack tip. This increases the amount of energy the aluminum and epoxy interface can absorb, increasing the peeling strength^31^. EDM texturing of aluminum substrate resulted in a peeling strength of 704.1 N/m, indicating a 191.9% increase compared to the untreated substrate. The craters and grooves on the substrate surface (see supplementary information, Fig. S3 f) are responsible for the increased peeling strength because they facilitate the mechanical interlocking of the epoxy resin and aluminum substrate. The epoxy gets embedded into the large craters formed due to electric discharge during EDM texturing. A strong mechanical interlock is formed upon curing between the aluminum substrate and epoxy. However, in comparison to the SAA specimen, the peel strength exhibited by the EDM textured specimen was lower by 44.1%. Although the EDM texture morphology increases the contact area between the two layers, creating a robust mechanical interlock becomes challenging because of the macroscopic craters.

### Lap shear test

**Fig. 7:**
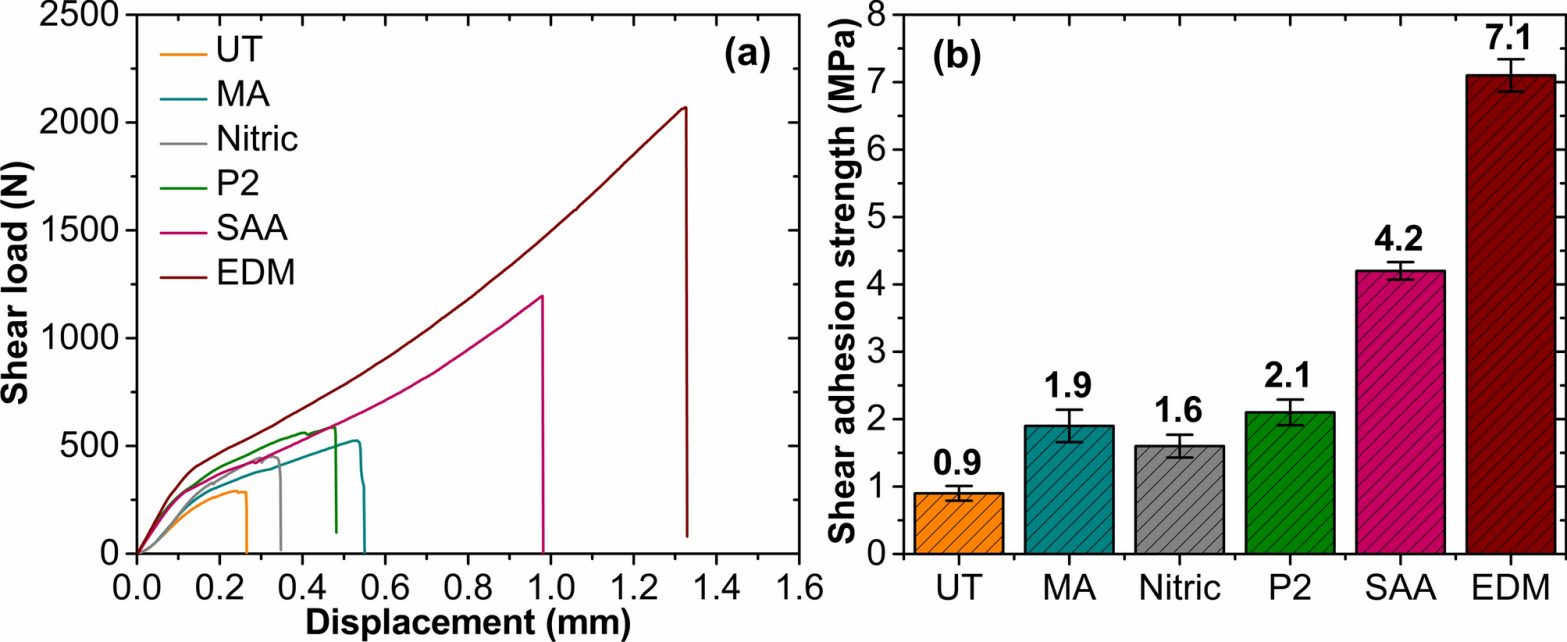
(**a**) Shear force versus displacement curves for specimens subjected to different surface treatments, (**b**) Shear adhesion strength for untreated and treated specimens.

Figure 7a presents the typical load–displacement curves for untreated and surface-treated specimens. As noted, specimens experience an abrupt failure, and the load drops abruptly from its maximum value. Compared with the untreated specimen, surface-treated specimens exhibited higher peak load and thus the shear strength.

Generally, the substrate subjected to various surface treatments showed substantial improvement in the adhesion shear strength (see Fig. 7b) compared to untreated specimens, displaying a shear strength of 0.95 MPa. The lower shear strength exhibited by the untreated specimen is ascribed to the poor wettability and weaker interface adhesion between the aluminum and CFRP. For specimens subjected to mechanical roughing, a 96.8% increase in shear adhesion strength was observed compared to the untreated one. The increase is attributed to the rough surface, which increases the contact area between the resin and aluminum increases, providing sites for the infiltrating epoxy resin to form mechanical interlocks. In comparison, the shear strength of chemically treated specimens increased by 66. 3% and 100%, respectively. The chemical treatments generated micro-pores, which act as sites for mechanical anchoring. The micro-pores enhance the surface energy and wettability of the treated substrates. As compared to the untreated specimen, higher roughness and surface energy in the case of chemical treatments result in a strong mechanical interlocking between the epoxy resin and substrates, thus improving the shear strength. The shear adhesion strength of the specimen subjected to SAA was 340% higher than that of the untreated specimen. Shear adhesion strength usually increases as surface roughness increases. However, this was not the case for specimens treated with SAA. Although the surface roughness of these specimens was noticeably lower than that of the specimens treated with other methods, the shear strength was noticeably higher. The main contributing factor was the uniformly distributed cylindrical nano-pores formed on the substrate surface. The porous oxide layer helped the SAA-treated specimen to have about 76.7% higher surface energy than the untreated specimen. The resin penetrated the porous layer (see supplementary information, Fig. S3 e) with enhanced surface energy and good wettability, thus increasing the intermolecular forces between the resin and substrate, which in turn strengthened the shear adhesion strength.

The specimen with the EDM-generated texture showed the greatest increase in shear adhesion strength. The shear strength of EDM textured specimen bettered the strength exhibited by SAA by 67.7%. The craters formed during the EDM texturing (see supplementary information, Fig. S3 f) act as sites for the epoxy to infiltrate and create a strong bond after curing. Since the load is applied in the plane normal to the infiltrations, the cured infiltrations offer very high resistance before failure. As a result, the shear peeling strength exhibited by the EDM textured specimen is higher compared to the other surface-treated specimens. The resistance to failure can be quantified from Fig. 7a, where the displacement before failure for EDM textured specimens is substantially higher than that of other surface-treated specimens. Also, it is to be noted that the EDM textured substrate exhibited poor wettability; however, a significantly higher force was needed to separate the specimen than the SAA-treated specimen. The air entrapment and surface tension of the water prevent the water droplets from quickly infiltrating the crater. However, since the curing was carried out at high pressure (4 bar), the resin is forced to penetrate the craters to form interlocks, thus increasing the bonding strength. Therefore, the application of contact angle and surface energy to represent bonding performance may be limited in the case of EDM textured surfaces.

### Fractographic analysis

**Fig. 8:**
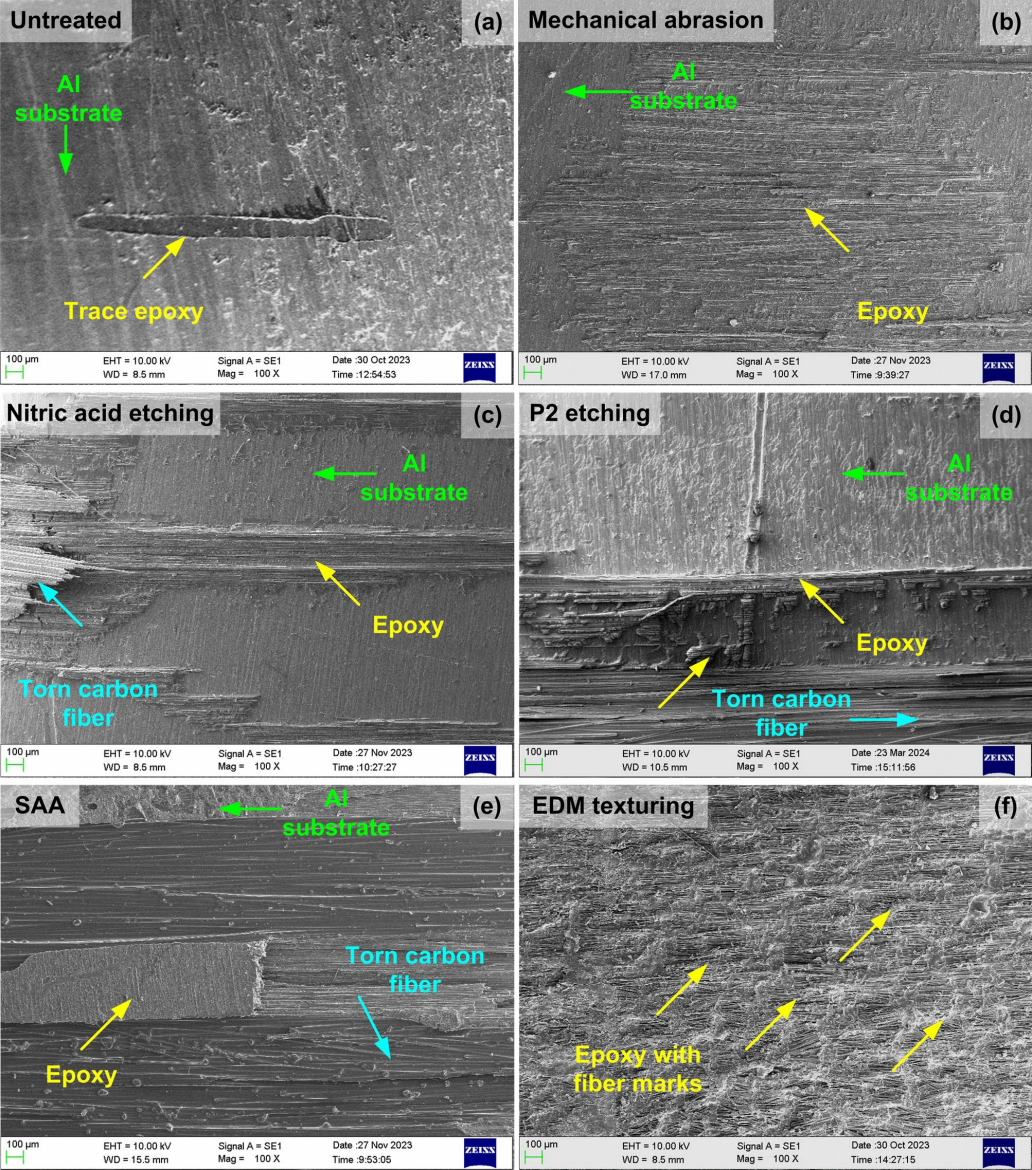
SEM images for the fractured substrate surface of (**a**) Untreated, (**b**) Mechanical abrasion, (**c**) Nitric acid etching, (**d**) P2 etching, (**e**) SAA, (**f**) EDM textured specimens.

Figure 8 shows the SEM images of the failed untreated and treated specimens after undergoing T-peel and shear peel tests, while the optical images can be visualized from supplementary information, Fig. S4.

As seen in Fig. 8a, the failed area of the untreated specimen is clean, with a trace amount of epoxy and no fiber adhesion. Failure mainly occurred at the aluminium-CFRP interface, indicating an adhesive failure. According to the failure mode, a weak bond between aluminum and CFRP makes it easy for cracks to form and spread when the external load is applied. Figure 8b displays the failed surfaces of specimens subjected to mechanical abrasion, a mixed mode failure (both adhesive and cohesive failures), with adhesive failure being the dominant mechanism. Moreover, the adherence of trace adhesive to the metal substrate collaborates with the improved shear strength offered by the abraded specimen compared to the untreated ones. Figures 8c, d show the delaminated substrate surface of specimens subjected to nitric acid and P2 etching. The surface indicated the presence of resin in some places, while certain locations lacked any resin, indicating a mixed mode failure (adhesive-cohesive failure). The presence of resin suggests an improvement in adhesion at the polymer-metal interface. In addition, CFRP adhesion and tear were observed on the failed surface. The fiber adhesion with the epoxy resin indicates a stronger bond with the metal substrate, which is beneficial to an increase in the peeling strength of the chemically treated specimens. Moreover, the improved adhesion exhibited in the case of P2 etching justifies the higher shear strength exhibited by the specimen compared to the nitric acid etched specimen. Figure 8e shows the fractured surface of the SAA-treated substrate after the test. The inspection of the surface revealed the presence of adhesion residue throughout the delaminated surface. In addition to the resin, carbon fibers were retained on the failed surface. This indicates that the adhesive adhered to the carbon fibers, which acted as an anchor and enhanced the bond strength. Overall, the strong, cohesive failure coupled with fiber adhesion suggested improved adhesion behavior and justified the enhanced peeling strength displayed by the SAA-treated specimens. Figure 8f shows the delaminated surface of the EDM textured specimen. In contrast to the mechanically abraded surface, the surfaces are covered with the resin material, indicating cohesive failure. The micro craters generated during EDM texturing acted as sites for the resin to infiltrate and form a strong bond upon curing. Additionally, fiber groove marks were visible, indicating some form of fiber adhesion before rupture. The strong, cohesive failure shown by the textured specimens is responsible for the significantly higher strength displayed by the EDM textured specimen.

## Conclusions

The presented study explored the various surface treatments (mechanical abrasion nitric acid etching, P2 etching, SAA, and EDM texturing) and their effects on the surface characteristics and interfacial strength of carbon fiber/epoxy and aluminum alloy-based FML. The findings led to the following conclusions:

- Surface treatments significantly influenced the surface roughness and surface energies of the aluminum substrate. EDM texturing resulted in large-sized crates, which increased the surface roughness (*S*_*a*_ = 7.61 μm); however, the surface showed hydrophobic behavior. Whereas SAA had a negligible effect on surface roughness (*S*_*a*_ = 0.14 μm) compared to other surface treatment methods, the SAA surface demonstrated the highest wettability of all the techniques employed.
- The peeling resistance and interfacial shear strength increased with the incorporation of surface treatment. SAA and EDM textured specimens exhibited better adhesion behavior than the abraded and chemically treated specimens. SAA and EDM texturing increased the T-peel strength of FMLs by 320.5% and 191.9%, while a 342.1% and 647.3% improvement in the shear peel strength was recorded compared to mechanically abraded specimens.
- Mechanically abraded aluminum substrate exhibited adhesive failure interfacial failure. The adhesion-cohesion (mixed mode) was the primary failure mode for chemical treatment methods, while the EDM textured surface exhibited a strong, cohesive failure behavior. Moreover, the SAA treatment demonstrated a mixed mode failure with fiber adhesion, indicating improved bonding strength in both Mode I and Mode II loading conditions.

## Supplementary Information

Supplementary Information.

## Data Availability

Data is provided within the manuscript and as supplementary information files.
