# Supplementary material for: Assessment of surface treatment methods for strengthening the interfacial adhesion in CARALL fiber metal laminates
Source: Sci Rep. 2024 Dec 28;14:30909. doi: 10.1038/s41598-024-81777-1 (PMC11681081; doi:10.1038/s41598-024-81777-1)
Supplement: Supplementary file 1 — Supplementary Information. [file 41598_2024_81777_MOESM1_ESM.pdf]

Supplementary information

Assessment of Surface Treatment Methods for Strengthening the Interfacial Adhesion in CARALL Fiber Metal Laminates

Madhusudhan Balkundhi [1], Satish Shenoy Baloor [2] and Gururaj Bolar [1,*]

[1] Department of Mechanical and Industrial Engineering, Manipal Institute of Technology, Manipal Academy of Higher Education, Manipal 576104, Karnataka, India

[2] Department of Aeronautical and Automobile Engineering, Manipal Institute of Technology, Manipal Academy of Higher Education, Manipal 576104, Karnataka, India

* Correspondence: gururaj.bolar@manipal.edu

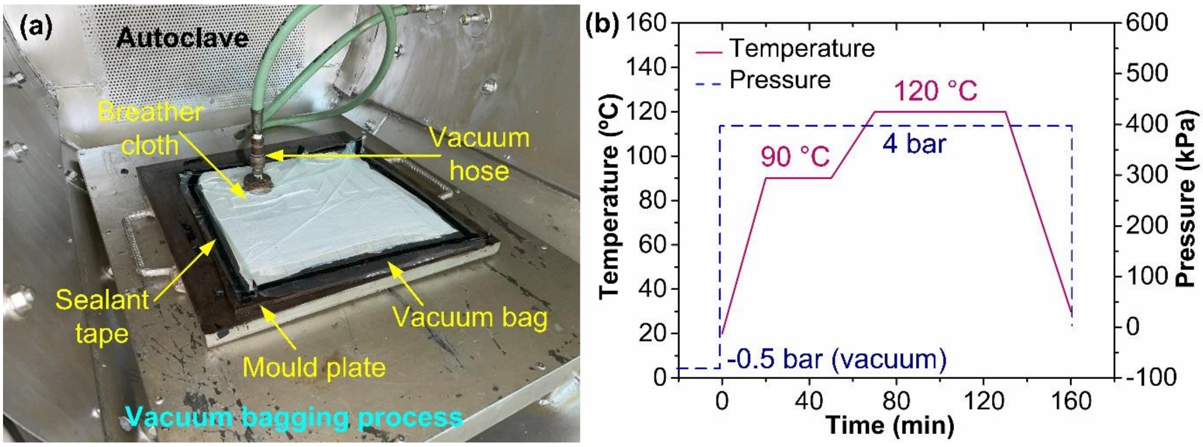

Fig. S1. (a) Vacuum bagging method to cure specimens inside autoclave, **(b)** Curing cycle employed in an autoclave.

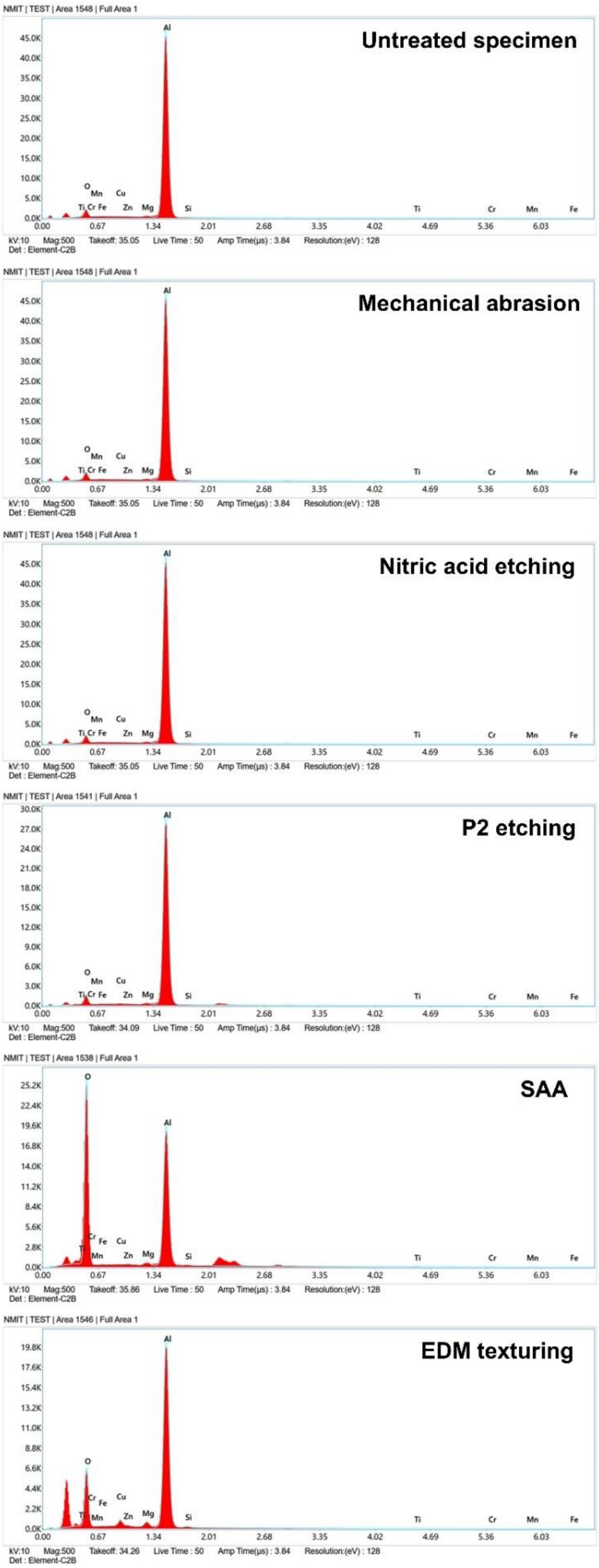

Fig. S2. EDS spectra for untreated and treated specimens**.**

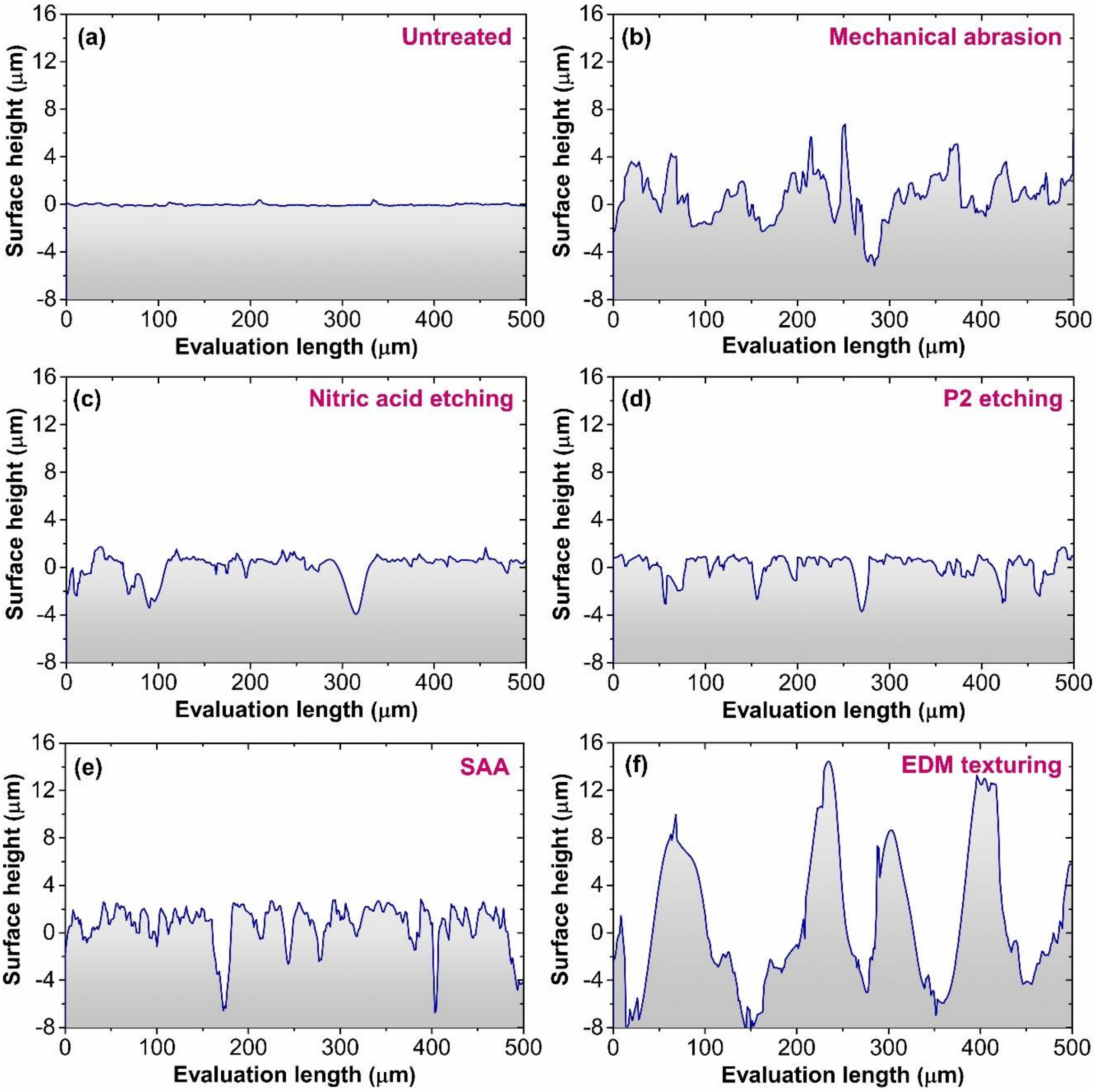

Fig. S3. Surface depth profiles of untreated and treated substrates.

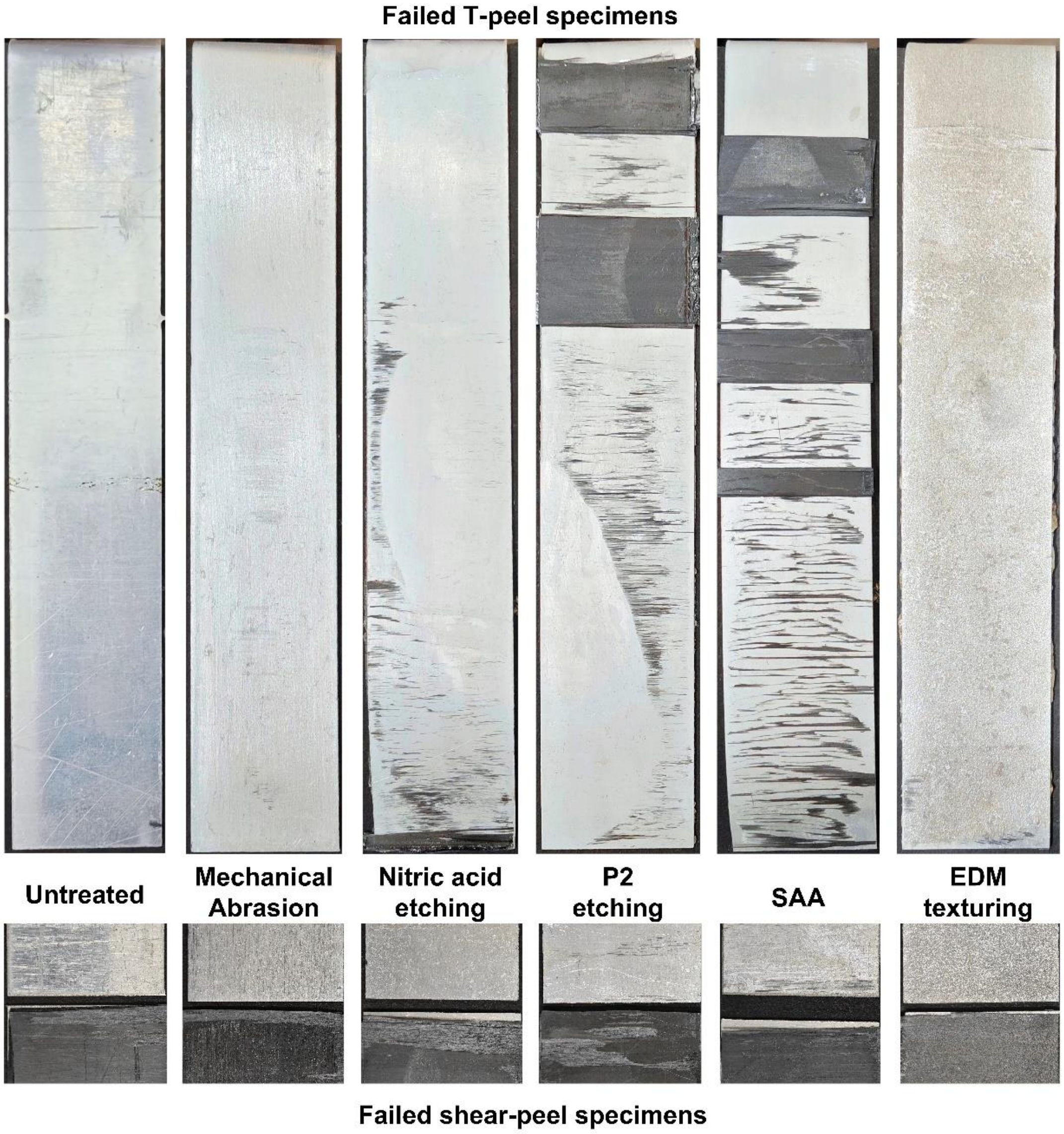

Fig.S4. Optical images of failed T-peel and shear-peel specimen surfaces subjected to different treatments.
